# Pluggers

**Published:** 1872-05

**Authors:** H. S. Chase


### ARTICLE X.

### *Pluggers.*

Nearly a year ago, at the meeting of the Illinois State Dental Society, we made our first public effort in favor of the smooth pointed plugger. Some time after we made another through the *Missouri Journal*, since then we have been watching to see what effect they would have upon the profession. They have at last reached the market—see the card from S. S. White, January, 1872, *Cosmos* There seems to be great fear of the smooth point slipping and possibly doing damage. Now, we maintain, as we have from the first, that this objection is groundless; we have used them for nearly two years and do not remember a single instance where we have been troubled by their slipping. The points we used were Atkinson's, Butler's, and latterly Varney's, with the serrations removed. We have heard of but one sound objection against their use, viz: "They leave the gold so bright that it is quite difficult to see where it is, and is not packed." Now, this objection is even more serious than those who advanced it suppose—their only objection was that it costs so much labor and care to properly condense the gold. We did not heed this because we were willing to give the extra labor and care, in order to have a more coherent filling than we conld make with the serrated point. In addition to this, we found in the course of time, that the glistening gold caused too constant a strain upon our eyes, for they pained us considerably, and it was very evident that we were overtasking them, and that seriously; we also had a terrible headache after every hard day's operating. These unpleasant results made us put the matter under serious consideration in order to find some way to overcome the difficulty. During a correspondence with Dr. C. Stoddard Smith, of Springfield, he suggested a broken point as preferable to either a smooth or serrated one, at the same time he rather condemned their use on account of the difficulty of their manufacture into the desired forms. We made one point and were well pleased with it, and believing "where there's a will there's a way," we went to work and made others, with more than satisfactory results.

Last year we were quite enthusiastic over the *smooth point*, and yet contend their infinite superiority over the finest serrated point, but we have nearly abandoned their use for the broken point. By the use of the broken point the gold is really frosted, and not punctured, as by the serrated point. If we could influence instrument manufacturers to stop the improvement and manufacture of the smooth point, it would prevent much dissatisfaction among those who use them, and doubtless be a saving of money to them, for we think in a few years this smooth point will be a drug in the market, having been superceded by the broken point. We want the manufacturers to present to the profession a set of instruments patterned after almost any set now in use, only, instead of the serrated points leave the grain of the steel after breaking,—Varney's instruments we consider the most perfect and simple—by doing this they will have produced instruments that will meet with more favor than any now in the market.

As far as we have used it, the broken point is perfect in its working, leaving the gold frosted or rather its surface quite dim, so at a glance we can tell where it is, and is not packed, and the roughness is so slight that it does not prevent a perfect cohesion, and last, and not least, we are troubled no more with headache, nor painful eyes. Nevertheless, we must with C. Stoddard Smith say, that at present the difficulty of their manufacture is an objection to them. It seems almost impossible to break the point perfect at the desired length, but it can be overcome. We have only tried it by breaking, but doubt not it can be accomplished by some chemical action upon the point, but be this as it may it can be done by breaking. The most difficult one to make is the *foot plugger.* It can be made by taking a bar of steel the size desired for the handle, the end desired for the point must be drawn down to a little larger, and about the shape desired for the face, harden to a full hard and break to a square break—it is often necessary to break two or three times before this is done—and then draw the temper to a soft, and shape the shank with a file, heat to a cherry red, place upon the heel and with a few gentle blows of the hammer applied to the instep the face is brought to the desired angle, then file to the shape wanted, and temper a little hard. As far as we know, it is necessary to first harden and then break the steel, in order to get the face, and work from that to get the instrument, using proper care not to injure the grain of the steel while it is soft.

We will not attempt to describe them any farther, or to tell how beautifully they work. As yet we know but comparatively little about them, but write this that others may try them and endeavor to devise some way to facilitate their manufacture. We know of several dentists who long ago knew of the broken point and used them to a considerable extent, but there are many who never heard of them, for such these remarks are intended.—*Missouri Dental Journal.*

